# Cognitive Function among Young Women's Football Players in the Summer Heat

**DOI:** 10.1155/2023/5516439

**Published:** 2023-11-02

**Authors:** Soichi Ando, Nana Ogoh, Shotaro Saito, Hironori Watanabe, Maki Ohsuga, Tetsuya Hasegawa, Shigehiko Ogoh

**Affiliations:** ^1^Graduate School of Informatics and Engineering, The University of Electro-Communications, Tokyo 182-8585, Japan; ^2^Chifure AS Elfen Saitama, Saitama, Japan; ^3^Kagawa Nutritional University, Sakado, Japan; ^4^Department of Biomedical Engineering, Toyo University, Kawagoe, Japan

## Abstract

Recently, there has been a growing focus on studies related to women's football. However, the cognitive function of female football players has not been extensively characterized. Thus, we explored how the cognitive function of female football players was altered during a series of matches in summer and examined day-to-day variations in cognitive function with regard to dehydration status. Resting cognitive function was assessed from 17 young women football players during the Japan Club Youth Women's football tournament, which spanned eight consecutive days. Cognitive function initially improved, with this improvement sustained throughout the tournament. It is worth noting that ten participants experienced symptoms of dehydration at least once during the tournament; however, these symptoms were not found to be linked to impaired cognitive function, suggesting that resting cognitive function remains unaffected during summer matches, even in the presence of dehydration symptoms.

## 1. Introduction

Growing attention has been devoted to studies on women's football [1–3]. During football matches, players face the challenges of making quick and accurate decisions in a constantly changing environment. Decision-making is a cognitive process that involves choosing a course of action from among several alternatives to achieve a goal. Hence, cognitive function is a key determinant of football performance. Intriguingly, research has shown that female and male players recruit different cognitive processes during football-specific cognitive tasks [4]. However, the cognitive functions of female football players have not been well characterized in the literature.

To succeed in a tournament, football players must maintain a good performance in a series of competitive matches. Recently, the level of environmental heat stress, which significantly impacts exercise performance [5], has been continuously rising due to a combination of increased prevalence, intensity, and duration of bouts of extremely hot weather [6]. Given that dehydration is frequently observed during exercise in hot environments [7], it is likely that football players will experience dehydration when playing consecutive matches in summer. This is a concern because dehydration may impair cognitive function [8]. Therefore, unless adequate hydration is restored during a tournament, cognitive function may be affected. Furthermore, although there is currently no evidence that women have an inherent disadvantage in thermoregulation when exercising in heat compared to men of similar age and health status [9], some studies have classified women as heat intolerant compared to men [10, 11]. In this context, it is particularly important to examine how the cognitive function of female football players is affected, especially when playing successive matches in the summer. Understanding the potential effects of dehydration on cognitive function will provide new insight into conditioning during consecutive summer matches.

In the present study, we investigated the impact of consecutive summer matches on the cognitive functions of female football players. Furthermore, we examined the day-to-day variations in cognitive function with respect to players' hydration status. The findings of this study provide practical information for women football players/coaches on how to preserve cognitive function during successive matches in hot summer conditions.

## 2. Materials and Methods

### 2.1. Participants

This study was conducted at the XF CUP 2021 Japan Club Youth (U18) women's football tournament. Participants were 17 young women football players (mean age, 17.2 ± 0.7 years). None of the participants had any cardiovascular, cerebrovascular, or respiratory diseases, and none were taking any prescribed or over-the-counter medications or supplements. This experimental protocol was approved by the Institutional Review Board of Toyo University (approval number: TU2021-022), and the participants (or parents/guardians) provided written informed consent prior to participation, in accordance with the principles of the Declaration of Helsinki, except for registration in a database.

### 2.2. Procedure

**Figure 1:**
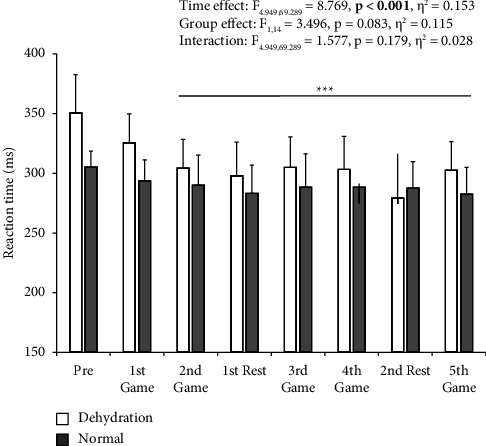
Reaction time in the Go trial of the Go/No-Go task during the tournament. White bars represent the dehydration group, and dark grey bars represent the normal group. Data are expressed as mean ± SD or median (interquartile range). ^*∗∗∗*^*p*  <  0.001 vs. pre. Note that the number of participants was six on the day of 2nd game in the normal group.

Cognitive function was assessed during the tournament (from the day before the first game (pregame) to the end of the fifth game) (Figure 1). The day before premeasurement, participants were familiarized with the cognitive task. Cognitive function was assessed on a daily basis in a serene hotel room, approximately 1-2 hours after dinner. Following the game, participants promptly returned to the hotel for rest. Consequently, cognitive function assessments were conducted more than 6 hours after the game. To verify the presence of dehydration, body weight and urine-specific gravity were measured. Importantly, the time elapsed between the assessment of cognitive function and body weight/urine measurements was consistently within 30 minutes. The participants provided a urine sample (100 ml), which was analysed for the concentration of excreted particles using a refractometer (PAL-09S; Shiro Sangyo, Osaka, Japan). Clinical dehydration was confirmed if the USG was ≥1.030 [12]. Based on these criteria, ten participants who experienced dehydration at least once during the tournament (once (*n* = 8), three times (*n* = 1), and six times (*n* = 1)) were classified in the dehydration group, while seven were classified into the normal group.

### 2.3. Cognitive Tasks

We used the Go/No-Go task to assess cognitive function [13]. This task is appropriate to evaluate decision-making in football players as it requires selective attention and response inhibition. Each trial began with a blank screen for 2.5 s, followed by a preparatory stimulus (green square) presentation at the centre of the computer screen for 1 s. Subsequently, one square of the four possible colours (red, blue, yellow, or purple) was presented for 1 s. One block of the Go/No-Go task consisted of 30 Go trials (red and blue) and 30 No-Go trials (yellow and purple). Participants responded to the Go signal by pressing the computer mouse with their right index finger as quickly as possible. Cognitive function was assessed using the reaction time (RT, ms) and accuracy (%). Omissions of responses in Go trials or incorrect responses in No-Go trials were regarded as error trials. Accuracy was calculated as the number of correct responses divided by the total number of trials.

### 2.4. Statistical Analysis

Statistical analyses were performed using JASP version 17.1 (JASP Team, Amsterdam, the Netherlands). Two-way repeated-measures analysis of variance was performed with day as the within-participant factor and group (dehydration vs. normal) as the between-participant factor. The degrees of freedom were corrected using the Huynh-Feldt epsilon when the assumption of sphericity was violated. Since there were missing data from one participant (RT and USG on the day of 2nd game), the data from that participant were excluded from the ANOVA. Post hoc analyses using Bonferroni corrections for multiple comparisons were applied, as appropriate. Additionally, we calculated separate averages of RT for both nondehydrated and hydrated days for each participant in the dehydrated group. Subsequently, we compared RT between nondehydrated and dehydrated days using a Wilcoxon signed-rank test. The effect sizes are presented as eta squares (*η*^2^). Data are expressed as mean ± SD for normal distributions or median (interquartile range) for nonnormal distributions. The significance level was set at *p*  <  0.05.

## 3. Results

### 3.1. Body Weight and Dehydration

We found a main effect of time on body weight (*F*_7,105_ = 4.620, *p*  <  0.001, *η*^2^ = 0.004) (Table 1). However, we did not observe any group difference (*F*_1,15_ = 3,927, *p*=0.066, *η*^2^ = 0.199) or interaction (*F*_7.105_ = 1.164, *p*=0.330, *η*^2^ = 0.001). Body weight was greater on the days of pre (*p*=0.019), 3rd game (*p*=0.019), 4th game (*p*=0.004), and 2nd rest (*p*=0.017) relative to the day of the 5th game. Body weight was also greater on the day of the 4th game than that 1st rest (*p*=0.027). As noted, ten participants exhibited dehydration at least once during the tournament. There was a main effect of the group on USG (*F*_1,14_ = 10.391, *p*=0.006, *η*^2^ = 0.153) (Table 1). However, there was neither a main effect of time (*F*_7,98_ = 1.500, *p*=0.176, *η*^2^ = 0.061) nor interaction (*F*_7,98_ = 0.307, *p*=0.949, *η*^2^ = 0.012).

### 3.2. Cognitive Function Changing during the Tournament

We observed a main effect of time on RT during the tournament (Figure 1). In contrast, we did not observe any group differences or interactions. RTs were shorter after the days of 2nd game compared to the prevalue (*p*  <  0.001). Additional analysis indicated that there were no differences in RT between nondehydrated (315 ± 23 ms) and dehydrated [290 (287–314) ms] days in the dehydrated group (*p*=0.193). Accuracy did not change during the tournament (*F*_1.514,22.711_ = 0.446, *p*=0.591, *η*^2^ = 0.022, main effect) (Table 1). There was neither a group difference (*F*_1,15_ = 0.194, *p*=0.666, *η*^2^ = 0.003, main effect) nor an interaction (*F*_1.514,22.711_ = 0.957, *p*=0.376, *η*^2^ = 0.047).

## 4. Discussion

In the current study, ten female football players exhibited symptoms of clinical dehydration (as indicated by a USG value of ≥1.030) at least once during the tournament, and the dehydration group exhibited higher USG levels throughout the tournament. However, it is important to note that changes in USG during the tournament did not exhibit differences between the two groups. A USG of 1.030 corresponds with a level of dehydration of 5% of body weight [14]. A recent meta-analysis study indicated that dehydration (>2% of body weight) induced by exercise with/without heat stress may impair cognitive function [8]. However, in the present study, cognitive function was not impaired throughout the tournament. Furthermore, within the dehydration group, no differences in RT were observed between nondehydrated and dehydrated days. This indicates that the onset of dehydration did not have detrimental effects on cognitive function. These results suggest that the resting cognitive function was well maintained under these experimental conditions. Rather, RT decreased on the days after the 2nd game as compared with prevalue, and the RT improvements persisted until the end of the tournament. Thus, the cognitive improvement is primarily attributed to the learning effect associated with repeat testing, which is inherent in prospective studies [15]. Although whether exercise-induced dehydration affects cognitive function remains controversial due to inconsistent methodological approaches [16, 17], acute and long-term improvements in hydration status have been suggested to be linked with better cognitive function [16]. Hence, our findings do not negate the need to prevent dehydration in soccer players to maintain cognitive function during summer matches. In addition, we assessed only resting cognitive function, and it remains unclear the extent to which the cognitive function of female football players is affected during football matches. Additional studies are needed to examine whether female football players' cognitive functions are affected during football matches in summer.

We acknowledge that there are several limitations inherent to the field study. First, we recognize that our sample size is relatively small and future studies with more participants are encouraged to draw firmer conclusions. Second, we matched the timing of cognitive assessment and body weight/urine measurements. However, all measurements were conducted at night. Hence, the timing of measurements could potentially influence an individual's level of alertness/arousal, which might, in turn, affect cognitive function. Third, it has been well-established that cognitive function is improved by acute exercise [18, 19]. In the present study, however, cognitive function assessments took place more than 6 hours after exercise. Meta-analysis studies have suggested that acute effects on cognitive function typically last for less than 30 minutes [20]. Therefore, it is less likely that acute exercise significantly affected cognitive function in the present study. Nevertheless, we acknowledge that there is a possibility of confounding factors that might have influenced the results. Finally, we did not verify the menstrual cycle phase of female football players. However, given that cognitive function was well-preserved throughout the tournament, these effects were presumably negligible.

## Figures and Tables

**Table 1:** Results of body weight, urine specific gravity (USG), and accuracy of the Go/No-Go task evaluation.

		Pre	1st game	2nd game	1st rest	3rd game	4th game	2nd rest	5th game
Body weight (kg)	Dehydration	56.0 ± 4.4	55.6 ± 4.3	55.7 ± 4.4	55.2 ± 4.6	55.9 ± 4.6	56.0 ± 4.8	56.0 ± 4.8	55.5 ± 4.9
	Normal	51.6 ± 4.8	51.5 ± 4.5	50.9 ± 4.4	51.1 ± 4.4	51.6 ± 4.5	51.8 ± 4.8	51.6 ± 4.6	50.5 ± 4.0
	All	54.2 ± 5.0^#^	53.9 ± 4.7	53.7 ± 4.9	53.5 ± 4.9	54.2 ± 5.0^#^	54.3 ± 5.1^†,##^	54.2 ± 5.1^#^	53.4 ± 5.1

USG	Dehydration	1.027 (1.018–1.028)	1.025 ± 0.008	1.028 ± 0.004	1.023 ± 0.008	1.025 ± 0.008	1.029 (1.020–1.030)	1.024 ± 0.011	1.021 ± 0.009
	Normal	1.021 ± 0.005	1.017 ± 0.009	1.019 ± 0.010, *n* = 6	1.015 ± 0.010	1.018 ± 0.005	1.020 ± 0.006	1.015 ± 0.009	1.013 ± 0.009
	All	1.025 (1.017–1.027)	1.022 ± 0.009	1.027 (1.023–1.029), *n* = 16	1.020 ± 0.010	1.022 ± 0.008	1.022 ± 0.009	1.020 ± 0.011	1.018 ± 0.009

Accuracy (%)	Dehydration	98.3 (98.3–100)	100 (97.3–100)	98.0 (97.0–100)	100 (97.3–100)	98.0 (97.0–98.0)	98.0 (97.0–100)	96.3 ± 2.8	98.0 (97.3–100)
	Normal	100 (96.7–100)	100 (98.5–100)	98.0 (96.0–98.0)	96.4 ± 3.5	96.6 ± 2.9	96.0 ± 4.1	96.0 ± 3.1	97.0 (94.5–97.0)

^†^ *p*  <  0.05 vs. 1st rest. ^#^*p*  <  0.05, ^##^*p*  <  0.01, vs. 5th game.

## Data Availability

The datasets are available from the corresponding author upon request.
